# Relationship of Smile Esthetics and Quality of Life Among High-School Adolescents in Al-Ahsa, Saudi Arabia: An Analytic Cross-Sectional Study

**DOI:** 10.3390/dj14010019

**Published:** 2026-01-02

**Authors:** Mohammed Alshaghdali, Syed Bokhari, Fatimah Bu Hulayqah, Yousef Almugla

**Affiliations:** 1 Hail Health Cluster, Ministry of Health, Hail 55471, Saudi Arabia; 2 Department of Preventive Dental Sciences, College of Dentistry, King Faisal University, Al-Ahsa 36362, Saudi Arabia; sbokhari@kfu.edu.sa (S.B.); yalmugla@kfu.edu.sa (Y.A.); 3 Al-Shefa Medical Polyclinic, Abqaiq 33261, Saudi Arabia; dr.fatimah.dentist@gmail.com

**Keywords:** adolescents, smile esthetics, oral health-related quality of life, PIDAQ, DESI, dental, psychosocial, Saudi Arabia

## Abstract

**Background/Objectives**: Adolescents may experience psychosocial consequences from minor dentofacial variations. The relationship between objectively rated smile esthetics and self-reported psychosocial impact remains under-studied in Saudi adolescents. This study aimed to investigate the relationship between the objectively measured smile esthetics with the subjectively reported psychosocial impact of perceived smile esthetics. **Methods**: Cross-sectional, multistage cluster-stratified sample technique was used to study adolescents aged 15–20 years (n = 344) from Al-Ahsa schools. Standardized extra-/intraoral photography supported Dental Esthetic Screening Index (DESI) scoring and psychosocial impact using Arabic Psychosocial Impact of Dental Aesthetics Questionnaire (PIDAQ) were applied. Reliability was assessed through two-way mixed intraclass correlation coefficient (ICC), Bland–Altman analysis, standard error of measurement (SEM), and minimal detectable change at the 95% confidence level (MDC95). Associations were examined using correlations and regression models. **Results**: The distribution of DESI categories was excellent (6.4%), good (29.7%), satisfactory (42.2%), insufficient (18.9%), and poor (2.9%). The distribution of PIDAQ impact levels was minimal (37.8%), slight (41.6%), moderate (18.0%), and significant (2.6%) (age *p* = 0.052; sex *p* = 0.417). DESI and total PIDAQ were weakly correlated (Spearman ρ = 0.248, 95% CI 0.143–0.347; *p* < 0.001). In a multivariable linear regression model with continuous PIDAQ total score as the outcome (R^2^ = 0.525; adjusted R^2^ = 0.516; *p* < 0.001), self-perceived smile dissatisfaction (B = 7.789; β = 0.478; *p* < 0.001) and tooth-color dissatisfaction (B = 4.099; β = 0.306; *p* < 0.001) showed the strongest associations with higher PIDAQ scores, while DESI total score showed a smaller association (B = 0.310; β = 0.120; *p* = 0.002). Age and sex were not significant predictors after adjustment. **Conclusions**: Objective smile esthetics were modestly associated with psychosocial impact, whereas adolescents’ self-perceived smile and tooth-color dissatisfaction were strongly associated with worse psychosocial outcomes. Although the smile esthetics may be clinically acceptable, adolescents can still experience reduced oral health-related quality of life due to the psychosocial impact of perceived dental esthetics. These findings support incorporating brief subjective questions on smile and tooth-color perception alongside objective assessment during routine adolescent dental care.

## 1. Introduction

Quality of life (QoL) is defined as a broad-ranging concept referring to an individual’s perception of their position in life that is influenced by one’s values, expectations, and environment, affecting one’s physical health, psychological state, social relationships, and functional condition [1,2,3]. The health aspect of QoL is termed as health-related quality of life (HRQoL) in clinical research [1]. Similarly, the QoL in relation to oral health is termed as oral health-related quality of life (OHRQoL) [3]. In medicine, the value of QoL has been widely acknowledged. However, QoL is a relatively new idea in dentistry, and oral health status has only been viewed in relation to QoL since the late 1980s [1]. Human diseases can have an impact on the quantity of life and/or the QoL. For some diseases, the quantity of years of life may not be reduced but various aspects of QoL may be affected. Lately, there is an increasing interest in numerous health conditions that may not be fatal but can still result in significant physical, social, and psychological dysfunction [4]. The QoL measurements have wide range of uses and applications, including a person’s psychosocial perception, satisfaction with care, expectations [2], extending the scope of outcome measures employed in clinical trials and other evaluative studies, evaluating the effectiveness of organizations or individuals, inspecting the health status of the community [1], and assessing the effectiveness and desirability of various treatment modalities [5]. Subjectively, the Psychosocial Impact Dental Aesthetics Questionnaire (PIDAQ) assesses the psychosocial impact of dental appearance and is validated in Arabic [6,7,8].

The smile is the most pleasing facial expression, expressing a person’s beauty, youth, and individuality [9]. The display zone of the smile is framed by the upper and lower lips. The teeth and the gingival scaffold are components of the smile inside this framework [10]. Regarding measuring smile esthetics, all common knowledge on dental esthetics is mainly based on experts’ writings which have been accepted by the Western populations [11]. The perception of smile esthetic is quite subjective, and this could be the reason why there are a few instruments developed with the aim of objectively quantifying and judging the smile esthetics. In the field of orthodontics, a few indices quantify esthetics, including the Index of Orthodontic Treatment Need (IOTN) and the Dental Aesthetic Index (DAI), and in the field of restorative, the Prosthetic Esthetic Index (PEI) has been developed to assess and quantify oral esthetics in patients receiving dental prosthetics [11]. The utilization of objective tools when assessing smile esthetics seems a good practice of standardization to allow for the possibility of future comparisons. Objectively, quantifying and judging the smile esthetics to a set of criteria by the clinicians is important and there are only few tools for this purpose, the Dental Esthetic Screening Index (DESI) is one of the most recently developed tools [11]. The DESI is a novel smile-specific and comprehensive objective assessment tool. Using objective indices alongside patient-reported outcomes may help clarify how clinician-rated esthetics relate to an individual’s perceived psychosocial impact.

The dental appearance, which includes the color, size, shape, position, and alignment of the teeth is regarded as a substantial determinant of attractiveness [12]. Any issue with the aforementioned characteristics could potentially impact the entire dentofacial esthetic. However, age group afflicted by dentofacial appearance issues is the younger age group, including children and adolescents, followed by adults [12]. Owing to the complexity of interactions between different aspects of an individual’s life, one aspect of life when negatively affected could affect the other aspects also, making changes in the quality of life. Despite growing interest in the relationship between esthetic indices and OHRQoL-related measures, evidence remains limited regarding how smile-specific objective esthetic ratings align with self-reported psychosocial impact in Saudi adolescents, and data from Al-Ahsa are scarce. Therefore, this study was focused on middle and late high-school adolescents to (i) profile objective smile esthetics, (ii) quantify psychosocial impact on QoL, (iii) test their association, and (iv) assess satisfaction with one’s smile in Al-Ahsa, as the literature search shows a scarcity of similar studies in the region.

## 2. Materials and Methods

### 2.1. Study Design and Setting

Cross-sectional analytical study on high-school adolescents in Al-Ahsa, Saudi Arabia, was conducted using a multistage cluster-stratified sampling technique. Boys’ and girls’ schools were selected randomly across administrative regions of Al-Ahsa (northern and eastern region, Al-Mubarrez, and Al-Hofuf).

### 2.2. Participants and Sampling Methodology

Middle and late adolescents aged 15 to 21 years living in Al-Ahsa, Saudi Arabia, were the target population of this study. Adolescents in this age group can be approached in high schools. According to the official statistical report of the General Authority for Statistics in the Kingdom of Saudi Arabia (GASTAT) published in 2022, the total population count of adolescents in Al-Ahsa city is around 150 thousand. According to the list of schools received from Management of Al-Ahsa Education, there are 200 high schools in Al-Ahsa, 103 male high schools and 97 female schools.

A multistage cluster-stratified sampling technique was employed. The first stage was to randomly select schools from each region of the city, stratified by gender (male and female schools). The second stage involved randomly selecting students from different grade levels and classrooms within each chosen school. Computer-generated randomization was used for both school and student selection. In total, 19 boys’ schools and 26 girls’ schools were selected and approached. Participation was obtained from 7 boys’ schools and 4 girls’ schools; the remaining selected schools either declined participation or could not be scheduled within the study period. Within participating schools, invited students who provided consent and completed both photography and questionnaires formed the analytic sample, yielding 270 boys and 74 girls (total n = 344).

Sample size calculation was based on n = *Z^2^P*(1 − *P*)/*d^2^* [13], giving an initial target of approximately 384 participants. The achieved sample was n = 344 (270 male; 74 female). The lower female participation primarily reflected refusal of photography (required for DESI scoring) due to local cultural norms and consent preferences, resulting in a male-skewed sample; the implications of this imbalance for precision and generalizability are addressed as a study limitation.

### 2.3. Tools and Data Collection

Objective esthetics were measured using the Dental Esthetic Screening Index (DESI; 10 items; total 10–50; lower = better) [11]. Categories were based on 8-point bands: 10–17 excellent, 18–25 good, 26–33 satisfactory, 34–41 insufficient, and 42–50 poor. These bands were used for interpretability rather than as clinically validated cut-offs. Subjective psychosocial impact was measured using the Arabic PIDAQ (23 items; 0–92; higher = worse) grouped into equal bands: 0–23 minimal, 24–46 slight, 47–69 moderate, and 70–92 significant [6,7]. For ordinal analyses, PIDAQ was categorized into four equal-width ranges (minimal, slight, moderate, significant). Because these cut-offs are not validated for clinical decision-making, additional analyses treating PIDAQ as continuous were conducted (Section 2.5).

A series of three photographs (Supplementary Figure S1) were taken for each participant (posed smile and retracted anterior view). Mouth retractors and reference rulers were used in the process of taking the photographs. A standardized technique was used in taking the photographs. The photographs were taken via two identical professional camera set-ups (Sony a6400 with Sony FE 85 mm F1.8 lens and Sony 128-GB memory card (Sony Corporation, Tokyo, Japan), Godox MF12 dental dual flash system with diffusers (Godox Photo Equipment Co., Ltd., Shenzhen, China), and tripod). Each one of the two data collectors (licensed general dentists) had one set-up, one male data collector went to the male high-schools, and the other was female who went to the female high-schools. Instructions followed during photographing were participant’s face has to be straight to the camera, participant’s head must be in neutral position no tilting forward nor backward, participant’s mouth distance to the camera lens to be in the range of 80 cm to 100 cm, and camera and participant’s mouth should be on the same level of height.

Participants’ demographics and smile satisfaction questionnaire were collected from each participant in which the questionnaire included a serial number which was unique for each participant instead of their name to preserve the privacy of participants as the first page of this questionnaire is to be photographed before the oral photos are taken. Demographics collected include age, gender, marital status, family financial level, work activity, satisfaction with smile esthetics question, liking the color of teeth question, and three dental history questions.

A protocol was developed with an orthodontist for the utilization of the DESI tool for the purpose of standardization and accuracy (Supplementary File S1). A single trained assessor performed all DESI analyses using the developed protocol.

### 2.4. Reliability

Intra-rater reliability was assessed using 36 cases randomly selected from the study sample and rescored after a 1-week interval by the same assessor. Reliability analyses included a two-way mixed-effects intraclass correlation coefficient (ICC; single- and average-measures), Bland–Altman analysis (mean difference and 95% limits of agreement), standard error of measurement (SEM), and minimal detectable change at 95% confidence (MDC95). Inter-rater reliability was not assessed because all DESI scoring was performed by a single assessor.

### 2.5. Analysis

Descriptives and cross-tabulations were applied by age (≤16 vs. >16) and gender (male/female). Bivariate associations used Spearman’s rank correlation coefficient (ρ) for DESI and PIDAQ total scores, and with subjective variables and dental-clinic history. Ordinal logistic regression (logit link) was initially fitted using PIDAQ impact categories as an ordered outcome to examine associations with DESI categories (simple model) and then with additional covariates (age, sex, prior dental-clinic visit, smile satisfaction, and tooth-color perception). The proportional odds assumption was evaluated using the test of parallel lines and was violated for the multivariable ordinal model. Collapsing PIDAQ into three ordered levels (minimal/slight, moderate, significant) did not resolve this violation (*p* < 0.001). Therefore, ordinal odds ratios are interpreted cautiously as approximate averaged associations across thresholds. To provide estimates not relying on the proportional odds assumption, multiple linear regression was additionally fitted with continuous PIDAQ total score as the dependent variable and DESI total score, age, sex, dental-clinic attendance, smile satisfaction, and tooth-color perception as predictors. Analyses were conducted on complete cases for DESI, PIDAQ and key covariates (n = 344). Statistical significance was set at α = 0.05. Analyses were conducted using IBM SPSS Statistics (version 30; IBM Corp., Armonk, NY, USA).

### 2.6. Ethics

Approvals were obtained from the Internal IRB (College of Dentistry, KFU) and KFU Research Ethics Committee (KFU-REC-2024-MAR-ETHICS2082; Approval Date: 20 March 2024); Education authority permission; and parental/guardian informed consent.

## 3. Results

Participants’ general characteristics are summarized in Table 1. The age range was 15–20 years (mean 16.47 ± 1.135); males comprised 78.5% of sample. Most participants reported low household income (≤10,000 SAR; 62.8%) and no paid work (93%). Dental-clinic attendance was 75.6%; anterior restorations, 36.6%; prior orthodontic treatment, 9.3%.

Smile satisfaction was clustered toward the positive/neutral range (Table 2, Panel A): satisfied/strongly satisfied, 48.0%, neutral, 30.5%, and dissatisfied/strongly dissatisfied, 21.5%, with no significant age/sex differences. Tooth-color satisfaction was skewed toward dissatisfaction (Table 2, Panel B), with 56.1% reporting “not at all”/“a little” satisfied; group differences were not significant.

The proportion of adolescents with ideal DESI scores for each smile component of the DESI tool are presented in Table 3; the only age-related signal was tooth/restoration-color (*p* = 0.042). Six items differed by sex (*p* ≤ 0.044), notably interdental papilla fill and arch continuity. Differences between males and females may be attributed to anatomical, developmental, or habitual factors. Full five-level item distributions of DESI are presented in Supplementary Table S1. Overall DESI categories (Table 4) were as follows: excellent: 6.4%, good: 29.7%, satisfactory: 42.2%, insufficient: 18.9%, and poor: 2.9%, with no significant subgroup differences.

PIDAQ impact categories (Table 5) were as follows: minimal: 37.8%, slight: 41.6%, moderate: 18.0%, and significant: 2.6% (age *p* = 0.052; sex *p* = 0.417). Correlations with total PIDAQ are shown in Table 6 and include the following: DESI (ρ = 0.248), smile dissatisfaction (ρ = 0.644), tooth-color dislike (ρ = 0.584), and prior dental-clinic visit (ρ = −0.143). Full PIDAQ items and subscales frequencies are presented in Supplementary Table S2 and associations by age and gender are presented in Supplementary Table S3.

In simple ordinal models (Table 7), excellent and good DESI categories were associated with lower odds of worse PIDAQ impact categories. In the multivariable ordinal model (Table 8), self-perceived smile dissatisfaction and tooth-color dissatisfaction showed the largest associations with PIDAQ impact, whereas DESI categories were no longer independently associated after adjustment. The proportional odds assumption for the multivariable ordinal model was violated (test of parallel lines *χ^2^*(30) = 199.152, *p* <0.001); therefore, ordinal odds ratios are interpreted as approximate averaged associations across thresholds. A sensitivity analysis using three PIDAQ levels showed similar patterns, but proportional odds remained violated (χ^2^(15) = 206.369, *p* < 0.001).

In multiple linear regression with continuous PIDAQ total score as the outcome (Table 9), the model accounted for 52.5% of the variance (*R^2^* = 0.525; adjusted *R^2^* = 0.516; *F*(6, 337) = 62.002; *p* < 0.001). Self-perceived smile dissatisfaction (B = 7.789, β = 0.478, *p* < 0.001; 95% CI 6.409–9.168) and tooth-color dissatisfaction (B = 4.099, β = 0.306, *p* < 0.001; 95% CI 2.972–5.227) were the strongest associated factors. DESI total score showed a smaller but statistically significant association (B = 0.310, β = 0.120, *p* = 0.002; 95% CI 0.113–0.506), whereas age and sex were not significant after adjustment (*p* > 0.20). Dental-clinic attendance showed a non-significant trend toward lower PIDAQ scores (B = −2.988, *p* = 0.064). Overall, conclusions were consistent across modeling approaches, with subjective perceptions showing substantially stronger associations with psychosocial impact than objective DESI scores.

DESI intra-rater (test–retest) reliability was high (two-way mixed-effects ICC, absolute agreement): single-measures ICC = 0.852 (95% CI 0.495–0.942) and average-measures ICC = 0.920 (95% CI 0.662–0.970), both *p* < 0.001. Bland–Altman analysis showed a mean difference of 2.36 units (95% limits of agreement −3.38 to 8.10); SEM was 2.07 and MDC95 was 5.74.

## 4. Discussion

Knowing and investigating the effects of oral diseases on quality of life is crucial to public health systems, research, and decision-making on strategies to be implemented in the prevention and promotion of oral health [14]. Smile esthetics can be affected by a plethora of oral diseases. Oral diseases affect the quality of life, and sometimes this effect is not limited to the person but extends beyond the individual’s life to the family and legal guardians as seen in the case of children and adolescents [15]. Regarding the age of adolescence the American Academy of Pediatrics (AAP) defines adolescence as spanning from 11 to 21 years of age, categorizing it as early (ages 11–14), middle (ages 15–17), and late (ages 18–21) adolescence [16]. Emotional instability peaks around 10–14 years [17]. This study, therefore, focused on middle and late adolescents found in high schools.

This study examined the relationship between the objectively rated smile esthetics (DESI) and psychosocial impact of dental appearance (Arabic PIDAQ) among high-school adolescents in Al-Ahsa. Overall, DESI and PIDAQ were weakly associated (ρ = 0.248), indicating that worse objective smile esthetics tended to coincide with higher psychosocial impact, but with a small effect size. Across multivariable analyses, subjective perceptions—particularly self-perceived smile dissatisfaction and tooth-color dissatisfaction—showed markedly stronger associations with psychosocial impact than objective DESI scores. In the linear regression model using continuous PIDAQ total score, self-perceived smile dissatisfaction (β = 0.478) and tooth-color dissatisfaction (β = 0.306) were the strongest associated factors, while DESI total score contributed to a smaller but statistically significant association (β = 0.120). This pattern suggests that adolescents’ perceptions capture a psychosocial burden beyond what is reflected by objective smile ratings alone. Clinically, this highlights that objective esthetic indices are informative but may not reliably identify adolescents experiencing psychosocial burden, reinforcing the need to incorporate patient perception into assessment and counseling.

Several methodological points are important for interpretation. First, DESI and PIDAQ categories were created using equal-width cut-offs to improve interpretability; these cut-offs are not validated clinical thresholds and may cause some boundary misclassification. To address this, we complemented categorical ordinal models with analysis using continuous PIDAQ total scores. Second, the proportional odds assumption for ordinal logistic regression was violated (test of parallel lines *p* < 0.001), even after collapsing PIDAQ categories, meaning that ordinal odds ratios should be interpreted as approximate averaged associations across thresholds rather than strictly constant effects. The linear regression model—which does not rely on the proportional odds assumption—provided robust supporting evidence and yielded a consistent overall pattern of findings, with subjective perceptions dominating psychosocial impact and DESI contributing only modestly. However, the present study adds to the literature by combining a smile-focused objective tool (DESI) with a validated Arabic PIDAQ in a Saudi adolescent population, helping to clarify the degree of alignment between clinician-rated smile esthetics and adolescents’ perceived psychosocial experience. Importantly, the weak DESI–PIDAQ association indicates that objective and subjective assessments should not be treated as interchangeable, rather, they provide complementary information.

In relation to the use of the relatively newly developed DESI tool [11], our literature review showed that the instrument has not been widely used, despite its good validity and reliability reported in the original development study. Slightly different findings were reported by a recent study conducted in Qassim University, Saudi Arabia, using the same tool (DESI) [18], in which the “good” category was the most common in their study (“satisfactory” in our study); however, the difference could be because the study only used the intraoral part of DESI tool, and the different age of participants (mean age 33.5 year), different approach of using DESI tool, lack of intra-reliability assessment, and the lack of mention of DESI category cut-off points used could also cause this difference. However, the same study has similar findings in which genders in relation to DESI scoring did not have statistically significant differences. Another study used the DESI tool [19] and compared three esthetic indices, namely IOTN-AC, DAI, and DESI, for planning orthodontic treatment, involving 242 participants (160 female, 82 male) aged 16–25 years interested in orthodontic care, the study reported that DESI showed high sensitivity but low specificity compared to IOTN-AC and DAI. However, the study mentioned an unrealistic average time of performing dental esthetic analysis using the DESI tool of around 68 s only, while in our study it took an average of approximately 15 min; moreover, the study reported that the DESI tool had 12 items while it has only 10 items [11] and the study did not report any reliability analysis when utilizing the DESI tool. Future studies using the DESI tool could benefit from our DESI protocol for the purpose of standardization (Supplementary File S1).

The literature search reported that the studies using the PIDAQ tool are describing the PIDAQ scores in comparison with other variables, in which they do not report the PIDAQ scores alone to convey what’s the perceived dental esthetics psychosocial impact status of participants. A Saudi study measured the psychosocial impact by the PIDAQ tool in comparison to the perception of presence of caries in the anterior teeth [20]; in their study sample (n = 72, age 18 and older) they found worse PIDAQ scores among participants who perceived the presence of caries. This is in line with our findings that the subjective self-reported variables have an impact on PIDAQ score. A different study on adolescents and young adults (n = 190, ages between 14 and 29, mean age 23) looked at the relationship between PIDAQ and the subjective oral health measured by the (OHIP-14) tool [21], and they found that the two variables are associated. This agrees with our findings that the subjective self-reported variables have an impact on PIDAQ score. Moreover, a study conducted in Brazil looking at PIDAQ, demographics, and clinical characteristics in their study sample (n = 505, mean age 36, 80% females) found that participants who dislike their smile, and who did not have prior dental esthetic treatment, have worse PIDAQ scores [22]; this is in agreement with our findings about dissatisfaction with smile in which it was associated with worse PIDAQ score.

### 4.1. Clinical Implications

The study recommends integration of subjective questions about smile and tooth-color satisfaction into dental screening alongside objective assessment to help identify those at higher psychosocial risk and support shared decision-making, counseling, and prioritization of esthetic interventions aligned with patient concerns. School esthetic literacy may normalize minor variations, reduce distress, and improve appropriate help-seeking.

### 4.2. Strengths

1. Use of concurrent objective (DESI) and subjective (Arabic PIDAQ) tools, 2. stratified sampling technique, 3. application of standardized, reliable DESI scoring (protocol developed for using DESI tool) with high intra-rater reliability, 4. adequate sample, and 5. multivariable modeling complemented by sensitivity analysis using continuous PIDAQ scores.

### 4.3. Limitations

The sample was male-skewed (270 boys vs. 74 girls) because participation required photography and, due to local cultural norms and consent preferences, many female adolescents and families declined imaging. This reduces precision for female estimates and introduces potential non-response bias if participating girls differ systematically from non-participants; generalization to female adolescents should therefore be made cautiously. The multistage cluster-sampling design (students nested within classes/schools) may also inflate sampling variance, and clustering was not explicitly modeled in regression analyses; standard errors may therefore be underestimated. The proportional odds assumption for ordinal logistic regression was violated, so ordinal odds ratios should be interpreted as approximate averaged associations. Finally, the study is cross-sectional; therefore, observed relationships should be interpreted as associations rather than causal effects.

## 5. Conclusions

In this cross-sectional study of adolescents in Al-Ahsa, objective smile esthetics (DESI) showed only a modest association with psychosocial impact, whereas self-perceived smile dissatisfaction and tooth-color dissatisfaction were strongly associated with worse PIDAQ scores. These findings support complementing objective esthetic assessment with brief subjective screening questions in adolescent dental care to better identify psychosocial needs and align counseling and treatment planning with patient priorities. Future studies with improved female participation and analytic approaches that account for clustering are recommended.

## Figures and Tables

**Table 1:** Participant characteristics and dental history (n = 344).

Domain	Metric/Category	Value
Age (years)	Range (min–max)	15–20
	Mean (SD)	16.47 (1.135)
Age distribution	15	77 (22.4%)
	16	105 (30.5%)
	17	105 (30.5%)
	18	41 (11.9%)
	19	12 (3.5%)
	20	4 (1.2%)
Gender	Male	270 (78.5%)
	Female	74 (21.5%)
Family income (SAR/month)	Low (≤10,000)	216 (62.8%)
	Medium (>10 k, <20 k)	103 (29.9%)
	High (≥20,000)	25 (7.3%)
Work activity	No work	320 (93.0%)
	Working	24 (7.0%)
Dental history	Ever visited dental clinic—Yes	260 (75.6%)
	Orthodontic treatment—Yes	32 (9.3%)
	Anterior restorations—Yes	126 (36.6%)

Note: Dental-clinic visit differed by gender (*χ^2^* = 6.075, *p* = 0.014); other age/sex splits non-significant.

**Table 2:** Satisfaction with smile and tooth color (overall; age; gender).

Panel A. Smile Satisfaction	
Level	n (%)
Strongly satisfied	55 (16.0%)
Satisfied	110 (32.0%)
Neutral	105 (30.5%)
Dissatisfied	54 (15.7%)
Strongly dissatisfied	20 (5.8%)
Group differences	Age χ^2^ = 1.441, *p* = 0.837; Gender χ^2^ = 7.107, *p* = 0.130
**Panel B. Tooth-Color Satisfaction**	
**Level**	**n (%)**
Not at all	124 (36.0%)
A little	69 (20.1%)
Somewhat	76 (22.1%)
Strongly	41 (11.9%)
Very strongly	34 (9.9%)
Group differences	Age *χ^2^* = 4.981, *p* = 0.289; Gender *χ^2^* = 4.580, *p* = 0.333

**Table 3:** Proportion of adolescents with ideal DESI scores by item, and associations with age and sex.

DESI Item	Ideal Appearance %	Age *p*-Value	Sex *p*-Value
Gingival contour	17.2	0.653	0.620
Interdental papilla	9.3	0.668	**0.007**
Arch continuity	12.5	0.078	**<0.001**
Angulation upper teeth	11.6	0.483	0.107
Proximal contacts	8.4	0.498	0.860
Tooth/restoration color	30.2	**0.042**	**0.020**
Central incisor W/H ratio	11.6	0.099	**<0.001**
Incisor–facial midline angle	47.4	0.150	**0.044**
Exposure of upper teeth by upper lip line	61.9	0.510	0.159
Lower lip line parallelism to smile line	46.8	0.913	**0.007**

Note: Ideal appearance refers to the DESI “ideal” category for each item. Percentages are based on the total sample (n = 344). Age and sex *p*-values are from *χ^2^* tests comparing category distributions across age group and sex. Numbers in bold font are statistically significant.

**Table 4:** DESI total category (overall; by age; by gender).

Category	Overall n (%)	≤16 (n = 182)	>16 (n = 162)	*p*-Value (Age)	Male (n = 270)	Female (n = 74)	*p*-Value (Sex)
Excellent	22 (6.4)	9 (2.6)	13 (3.8)	0.314	18 (5.2)	4 (1.2)	0.364
Good	102 (29.7)	60 (17.4)	42 (12.2)		77 (22.4)	25 (7.3)	
Satisfactory	145 (42.2)	78 (22.7)	67 (19.5)		110 (32.0)	35 (10.2)	
Insufficient	65 (18.9)	29 (8.4)	36 (10.5)		56 (16.3)	9 (2.6)	
Poor	10 (2.9)	6 (1.7)	4 (1.2)		9 (2.6)	1 (0.3)	

**Table 5:** PIDAQ impact category (overall; by age; by gender).

Category	Overall n (%)	≤16 (n = 182)	>16 (n = 162)	*p*-Value (Age)	Male (n = 270)	Female (n = 74)	*p*-Value (Sex)
Minimal	130 (37.8)	73 (21.2)	57 (16.6)	0.052	103 (29.9)	27 (7.8)	0.417
Slight	143 (41.6)	81 (23.5)	62 (18.0)		111 (32.3)	32 (9.3)	
Moderate	62 (18.0)	26 (7.6)	36 (10.5)		47 (13.7)	15 (4.4)	
Significant	9 (2.6)	2 (0.6)	7 (2.0)		9 (2.6)	0 (0.0)	

**Table 6:** Correlations with total PIDAQ (n = 344).

Pair	Spearman ρ	95% CI	*p*-Value	Magnitude, Direction
PIDAQ ↔ DESI total	0.248	0.143–0.347	<0.001	Weak, positive
PIDAQ ↔ Smile dissatisfaction	0.644	0.575–0.704	<0.001	Strong, positive
PIDAQ ↔ Prior clinic visit	−0.143	−0.248 to −0.034	0.008	Weak, negative
PIDAQ ↔ Tooth-color dissatisfaction	0.584	0.507–0.651	<0.001	Moderate, positive

**Table 7:** Simple ordinal logistic regression: DESI → PIDAQ category.

DESI Category (vs Poor)	OR (95% CI)	*p*-Value
Excellent	0.21 (0.05–0.87)	**0.031**
Good	0.25 (0.07–0.84)	**0.025**
Satisfactory	0.38 (0.12–1.25)	0.112
Insufficient	0.81 (0.24–2.76)	0.733

Model: *χ^2^*(4) = 20.21, *p* < 0.001; Nagelkerke *R^2^* = 0.064; reference = poor DESI. Numbers in bold font are statistically significant.

**Table 8:** Multivariable ordinal logistic regression predicting PIDAQ category.

Predictor	OR (95% CI)	*p*-Value
Age (per year)	1.23 (1.00–1.50)	**0.045**
Smile dissatisfaction	3.21 (2.47–4.18)	**<0.001**
Prior dental-clinic visit	0.54 (0.32–0.92)	**0.023**
Tooth-color dissatisfaction	1.85 (1.50–2.28)	**<0.001**
Gender (male vs. female)	1.19 (0.68–1.91)	0.540
DESI excellent vs. poor	0.39 (0.08–1.92)	0.245
DESI good vs. poor	0.84 (0.22–3.24)	0.805
DESI satisfactory vs. poor	0.82 (0.22–3.05)	0.765
DESI insufficient vs. poor	2.17 (0.56–8.41)	0.263

Model fit: *χ^2^*(9) = 217.36, *p* < 0.001; Nagelkerke *R^2^* = 0.522. Numbers in bold font are statistically significant.

**Table 9:** Multiple linear regression predicting total PIDAQ score.

Predictor	B (95% CI)	Standardized β	*p*-Value
Age (per year)	0.766 (−0.421 to 1.952)	0.048	0.205
Smile dissatisfaction	7.789 (6.409 to 9.168)	0.478	**<0.001**
Prior dental-clinic visit	−2.988 (−6.151 to 0.174)	−0.072	0.064
Tooth-color dissatisfaction	4.099 (2.972 to 5.227)	0.306	**<0.001**
Gender (male vs. female)	−1.197 (−4.480 to 2.087)	−0.027	0.474
DESI total score	0.310 (0.113 to 0.506)	0.120	**0.002**

Model fit: *R* = 0.724; *R^2^* = 0.525, Adjusted *R^2^* = 0.516, *F*(6,337) = 62.00, p < 0.001. Numbers in bold font are statistically significant.

## Data Availability

De-identified data and the DESI scoring protocol are available from the corresponding author upon reasonable request, subject to institutional approvals.

## Supplementary Materials

The following supporting information can be downloaded at: https://www.mdpi.com/article/10.3390/dj14010019/s1, Table S1: DESI item-level frequencies and associations by age and gender; Table S2: PIDAQ item frequencies (overall, n = 344); Table S3: PIDAQ item-level group comparisons (*χ^2^ p*-values); Figure S1: Example of the series of photographs of the participants; File S1: Protocol for using DESI smile analysis tool.

## Abbreviations

The following abbreviations are used in this manuscript:

DESI	Dental Esthetic Screening Index
PIDAQ	Psychosocial Impact of Dental Esthetics Questionnaire
ICC	Intraclass correlation coefficient
SEM	Standard error of measurement
MDC	Minimal detectable change
CI	Confidence interval
OR	Odds ratio
QoL	Quality of life
HRQoL	Health-related quality of life
OHRQoL	Oral health-related quality of life
IOTN	Index of Orthodontic Treatment Need
DAI	Dental Aesthetic Index
PEI	Prosthetic Esthetic Index
AAP	American Academy of Pediatrics
GASTAT	General Authority for Statistics in Saudi Arabia
KFU	King Faisal University
χ^2^	Chi-square
LoA	Limits of agreement
IOTN-AC	Index of Orthodontic Treatment Need, Aesthetic Component
OHIP-14	Oral Health Impact Profile 14-item version
IOTN-DHC	Index of Orthodontic Treatment Need, Dental Health Component
