# Supplementary material for: Relationship of Smile Esthetics and Quality of Life Among High-School Adolescents in Al-Ahsa, Saudi Arabia: An Analytic Cross-Sectional Study
Source: Dent J (Basel). 2026 Jan 2;14(1):19. doi: 10.3390/dj14010019 (PMC12839607; doi:10.3390/dj14010019)
Supplement: Supplementary file 1 [file dentistry-14-00019-s001.zip › dentistry-4014713-supplementary.pdf]

## Supplementary Tables S1–S3, Figure S1 and File S1

**Supplementary Table S1.** DESI item-level frequencies and associations by age and gender

| DESI item | Category | n (%) | Age p-value | Sex p-value |
|---|---|---|---|---|
| Gingival contour | Ideal | 69 (17.2) | 0.653 | 0.620 |
| | 1 tooth deviating | 45 (13.1) | | |
| | 2 teeth deviating | 109 (31.7) | | |
| | 3 teeth deviating | 71 (20.6) | | |
| | ≥4 teeth deviating | 60 (17.4) | | |
| Interdental papilla | Filled | 32 (9.3) | 0.668 | **0.007** |
| | 1 Not filled | 27 (7.8) | | |
| | 2 Not filled | 72 (20.9) | | |
| | 3 Not filled | 80 (23.3) | | |
| | ≥4 Not filled | 133 (38.7) | | |
| Arch continuity | Continuous | 43 (12.5) | 0.078 | **<0.001** |
| | 1 gap | 37 (10.8) | | |
| | 2 gaps | 72 (20.9) | | |
| | 3 gaps | 81 (23.5) | | |
| | ≥4 gaps | 111 (32.3) | | |
| Angulation upper teeth | Ideal / | 40 (11.6) | 0.483 | 0.107 |
| | 1 tooth deviating | 79 (23.0) | | |
| | 2 teeth deviating | 83 (24.1) | | |
| | 3 teeth deviating | 72 (20.9) | | |
| | ≥4 teeth deviating | 70 (20.3) | | |
| Proximal contacts (PC) | Ideal / | 29 (8.4) | 0.498 | 0.860 |
| | 1 PC deviating | 44 (12.8) | | |
| | 2 teeth deviating | 74 (21.5) | | |
| | 3 teeth deviating | 72 (20.9) | | |
| | ≥4 teeth deviating | 125 (36.3) | | |
| Tooth/restoration color | Harmonious | 104 (30.2) | **0.042** | **0.020** |
| | 1 tooth deviating | 58 (16.9) | | |
| | 2 teeth deviating | 115 (33.4) | | |
| | 3 teeth deviating | 38 (11.0) | | |
| | ≥4 teeth deviating | 29 (8.4) | | |
| Central incisor W/H ratio | Ideal | 40 (11.6) | 0.099 | **<0.001** |
| | ±0.05 | 128 (37.2) | | |
| | ±0.1 | 100 (29.1) | | |
| | ±0.15 | 31 (9.0) | | |
| | ≥±0.2 | 45 (13.1) | | |
| Incisor–facial midline angle | Parallel | 163 (47.4) | 0.150 | **0.044** |
| | ±5° | 99 (28.8) | | |
| | ±10° | 56 (16.3) | | |
| | ±15° | 17 (4.9) | | |
| | ≥20° | 9 (2.6) | | |
| Exposure of Upper Teeth by Upper lip line | Ideal | 213 (61.9) | 0.510 | 0.159 |
| | ±2mm | 72 (20.9) | | |
| | ±4mm | 45 (13.1) | | |
| | ±6mm | 13 (3.8) | | |
| | ≥8mm | 1 (0.3) | | |
| Lower lip line parallelism to | Convex-Parallel | 161 (46.8) | 0.913 | **0.007** |

| | | |
|---|---|---|
| smile line | Straight | 61 (17.7) |
| | Oblique | 79 (23.0) |
| | Minimally concave / | 18 (5.2) |
| | Distinctively concave | 25 (7.3) |

Note: Percentages are based on the total sample (n = 344). Age and sex *p*-values are from $\chi^2$ tests comparing category distributions across age group and sex. Numbers in bold font are statistically significant.

**Supplementary Table S2.** PIDAQ item frequencies (overall, n=344)

**Panel A.** Dental Self-confidence subscale (order: Very strongly, Strongly, Somewhat, A little, Not at all)

| Item | VS | S | SW | AL | NA |
|---|---|---|---|---|---|
| 1. Proud of my teeth | 49 | 69 | 119 | 60 | 47 |
| 2. Like to show teeth when smiling | 55 | 55 | 81 | 94 | 59 |
| 3. Satisfied with teeth in mirror | 44 | 56 | 97 | 83 | 64 |
| 4. Teeth attractive to others | 32 | 28 | 77 | 79 | 128 |
| 5. Satisfied with appearance of teeth | 60 | 52 | 82 | 76 | 74 |
| 6. Like arrangement/regularity | 54 | 46 | 70 | 75 | 99 |
| **Panel B.** Social impact subscale (order: Not at all, A little, Somewhat, Strongly, Very strongly) | | | | | |
| **Item** | **NA** | **AL** | **SW** | **S** | **VS** |
| 7. Back up when smiling | 138 | 97 | 61 | 29 | 19 |
| 8. Worried about unknowns' opinion | 164 | 77 | 67 | 11 | 25 |
| 9. Afraid of bad comments | 172 | 83 | 34 | 31 | 24 |
| 10. Decrease social contact | 243 | 56 | 20 | 12 | 13 |
| 11. Hand in front to hide teeth | 211 | 73 | 29 | 12 | 19 |
| 12. Feel people stare at teeth | 200 | 71 | 32 | 24 | 17 |
| 13. Comments bother me | 186 | 71 | 33 | 33 | 21 |
| 14. Worry about opposite gender opinion | 197 | 67 | 40 | 19 | 21 |
| 15. Others have more beautiful teeth | 109 | 91 | 62 | 36 | 46 |
| **Panel C.** Esthetic attitude subscale (order: Not at all, A little, Somewhat, Strongly, Very strongly) | | | | | |
| **Item** | **NA** | **AL** | **SW** | **S** | **VS** |
| 16. Envy people with beautiful teeth | 239 | 48 | 25 | 14 | 18 |
| 17. Frustrated when seeing others' teeth | 214 | 80 | 23 | 13 | 14 |
| 18. Unhappy with appearance of teeth | 139 | 102 | 49 | 29 | 25 |
| 19. Feel bad when thinking about teeth | 167 | 91 | 41 | 21 | 24 |
| 20. Wish teeth looked better | 52 | 50 | 36 | 52 | 154 |
| 21. Don’t like to see teeth in mirror | 181 | 88 | 38 | 18 | 19 |
| 22. Don’t like to see teeth in pictures | 163 | 70 | 53 | 24 | 34 |
| 23. Don’t like to see teeth in videos | 181 | 66 | 46 | 21 | 30 |

**Supplementary Table S3.** PIDAQ item-level group comparisons ($\chi^2$ *p*-values)

| Item | Age (≤16 vs >16) | Gender (M vs F) | Note |
|---|---|---|---|
| 5. Satisfied with appearance of teeth | ns | 0.036 | Higher dissatisfaction in males |
| 11. Hand in front to hide teeth | ns | 0.003 | Higher in males |
| 12. Feel people stare at teeth | 0.003 | ns | Higher in older adolescents |
| 17. Frustrated when seeing others' teeth | 0.017 | ns | Higher in older adolescents |

Note: All other PIDAQ items: no significant differences by age or gender at $\alpha$=0.05.

**Supplementary Figure S1.** Example of the Series of Photographs of the Participants

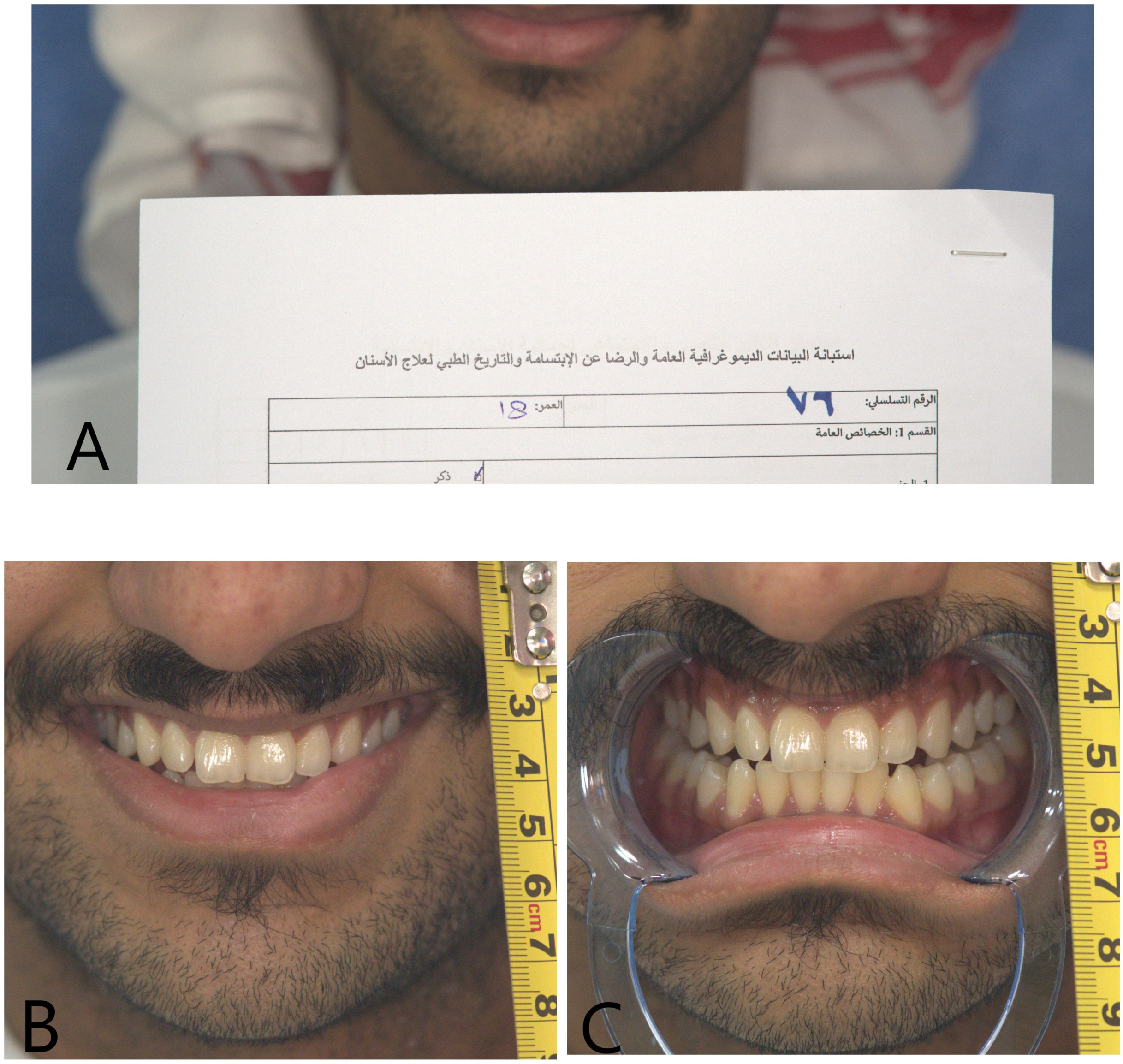

**Supplementary File S1.** Protocol for Using DESI Smile Analysis Tool

- In case of asymmetrical discrepancy is present between left and right side, the more deviating side will be considered in the analysis.
- Photos to be adjusted (by rotation) to make the facial midline vertical which determined by the eyes or eye shadows, nose, and chin.
- Intraoral:
  - 1. Gingival contour:
    - Each group of central, lateral and canine on either side is scored separately.
    - Canines' zenith points to be higher than centrals' zenith points by **maximum** 1 mm or at zenith of centrals is accepted, more than 1 mm is considered deviating.
    - Canines' zenith points lower than centrals by **more than 1 mm** considered deviating.
    - Zenith of laterals to be lower than centrals' zenith points by between 0.5 and 1 mm, less than 0.5 mm or more than 1 mm, the tooth considered deviating.
    - If the zeniths of the central incisors are on different levels, 1 tooth deviance is counted.
    - Score 1= ideal, score 2= 1 tooth deviating, score 3= 2 teeth deviating, score 4= 3 teeth deviating, score 5= 4 or more teeth deviating.
  - 2. Interdental space (IS):
    - Presence of black triangles in between the teeth even if extremely small, the space is considered not filled.
    - Presence of gap considered not filled.
    - Score 1= IS completely filled, score 2= 1 IS not filled, score 3= 2 IS not filled, score 4= 3 IS not filled, score 5= 4 or more IS not filled.
  - 3. Continuity of upper arch:
    - For crowding, each case to be assessed mesially and distally, 1 tooth could be scored as 2.
    - Score 1= continuous dental arch (no gaps or crowding), score 2= 1 gap or crowding, score 3= 2 gaps and/or crowdings, score 4= 3 gaps and/or crowdings, score 5= 4 or more gaps and/or crowdings.
  - 4. Angulation of upper teeth:
    - Parallel or slight mesial inclination of crowns of centrals is accepted. **Distal inclination** is considered deviating.
    - Mesial inclination for lateral and canines is ideal. **Parallel and distal** inclination are considered deviating.
    - Facial midline is to be used for reference.
    - Score 1= ideal relation, score 2= 1 tooth deviating, score 3= 2 teeth deviating, score 4= 3 teeth deviating, score 5= 4 or more teeth deviating.
  - 5. Position of proximal contacts (PC):
    - The middle point from beginning of teeth touching to the first point of inter dental papilla between the teeth is to be considered the point of contact PC.
    - Beginning with the PC of centrals and continuing distally, the contact points to be elevating apically as going distally considered ideal.
    - **PCs missing or going coronally or on a straight line to the mesial PC,** are considered deviating.
    - Score 1= PCs ideal, score 2= 1 PC deviating/missing, score 3= 2 PCs deviating/missing, score 4= 3 PCs deviating/missing, score 5= 4 or PCs deviating/missing.
  - 6. Tooth color and color of restorations harmony:
    - First the dominant color among the 6 front teeth (incisors and canines) is identified and considered as the normal color. Then teeth with colors that deviate from this dominant color are counted.
    - If a single tooth has multiple distinct colors (due to fluorosis, plaque, caries, or stains), that tooth is considered deviating.
    - Score based on number of deviating teeth: 0 teeth=score 1 (perfect color harmony), 1 tooth=score 2 (minor variations), 2 teeth=score 3 (moderate variations), 3 teeth=score 4 (significant variations), 4+ teeth=score 5 (severe disharmony). If all 6 teeth each have multiple distinct colors, all are considered deviating, resulting in score 5.
  - 7. Width-to-Height Ratio of Upper Central Incisor:
    - Ideal Condition: Width-to-height ratio of 0.8 (80%)
    - Measure width and height of the most deviated central incisor
    - Calculate width-to-height ratio (Width/height).

- Scoring: Score 1: Ratio of 0.8 (ideal), Score 2: Ratio of 0.75 or 0.85, Score 3: Ratio of 0.7 or 0.9, Score 4: Ratio of 0.65 or 0.95, Score 5: Ratio of ≤0.6 or ≥1.0.

- Extra-oral:
  - 1. Relation Between Angulation of Upper Central Incisors and Facial Midline:
    - Most extreme rotated central incisor is to be considered if any is rotated.
    - Score 1 = central incisors are parallel to the facial midline; Score 2 = one of them (centrals) is at least 5 degrees rotated to the facial midline; Score 3 = one of them is at least 10 degrees rotated to the facial midline; Score 4 = one of them is at least 15 degrees rotated to the facial midline; Score 5 = one of them is at least 20 degrees rotated to the facial midline. Always use the most deviating central incisor for scoring.
  - 2. Exposure of upper front teeth by the upper lip during smiling (upper lip line):
    - The most extreme point from **any of the 4 incisors** deviating either positive or negative to be used for the measurement.
    - If all teeth are shown and some of the gingiva then the gingiva to the lip will be measured and if the numbers are in positive 0 to <2mm score 1, ≥2mm score 2, ≥4mm score 3, ≥6mm score 4 and ≥8mm score 5.
    - If zenith points of any of the 4 incisors are covered by the upper lip then the measurement will be by subtraction of what length is shown during smile from the full length of the incisor taken from the photo with the retractors, and if what is show is < 70% of the full length (full length in mm is multiplied by 0.7) by < 2mm then considered in score 1, ≥ 2mm is score 2, ≥ 4mm then score 3 is given, ≥ 6mm then score 4 is given, ≥ 8mm then score 5 is given.
  - 3. Parallelism lower lip line to the dental arch of upper teeth (Smile line):
    - **Tips of canine** are the points for the measurement of curvature.
    - If NOT shown, consider **Laterals, or what is shown**.
    - If no teeth edges are shown, then score 5 (extremely deviated) is given.
    - If the canines or laterals or the most separated teeth shown (priority to canines, then laterals then centrals) are connected by a line and the centrals' edges are below it then convex, if centrals are above the line then concave, if above it by 2 or more mm then distinctively concave, if on the line then straight, if the most separated teeth shown are not on the same line ( and other teeth showing convex or straight pattern) one of them is deviated then oblique to be chosen.
